# Examination of Candidates for Assistant Surgeon

**Published:** 1914-08

**Authors:** 


### EXAMINATION OF CANDIDATES FOR ASSISTANT SURGEON.

TREASURY DEPARTMENT, UNITED STATES PUBLIC HEALTH SERVICE.

Washington, August 25, 1914.

Boards of commissioned medical officers will be convened to meet at the Bureau of Public Health Service, 3 B Street, SE., Washington, D. C., and at the Marine Hospitals of Boston, Mass., Stapleton, N. Y., Chicago, Ill., St. Louis, Mo., New Orleans, La., and San Francisco, Cal., on Monday, October 19, 1914, at 10 o'clock a. m., for the purpose of examining candidates for admission to the grade of assistant surgeon in the Public Health Service, when applications for examination at these stations are received in the Bureau.

Candidates must be between 23 and 32 years of age, graduates of a reputable medical college, and must furnish testimonials from two responsible persons as to their professional and moral character. Service in hospitals for the insane or experience in the detection of mental diseases will be considered and credit given in the examination. Candidates must have had one year's hospital experience or two years' professional work.

Candidates must be not less than 5 feet, 4 inches, nor more than 6 feet, 2 inches, in height.

The following is the usual order of the examinations: 1, Physical; 2, Oral; 3, Written; 4, Clinical.

In addition to the physical examination, candidates are required to certify that they believe themselves free from any ailment which would disqualify them for service in any climate and that they will serve wherever assigned to duty.

The examinations are chiefly in writing, and begin with a short autobiography of the candidate. The remainder of the written exercise consists of examination in the various branches of medicine, surgery, and hygiene.

The oral examination includes subjects of preliminary education, history, literature, and natural sciences.

The clinical examination is conducted at a hospital.

The examination usually covers a period of about ten days.

Successful candidates will be numbered according to their attainments on examination, and will be commissioned in the same order. They will receive early appointments.

After four years' service, assistant surgeons are entitled to examination to the grade of passed assistant surgeon.

Assistant surgeons receive $2,000, passed assistant surgeons $2,400, surgeons $3,000, senior surgeons $3,500, and assistant surgeon generals $4,000 a year. When quarters are not provided, commutation at the rate of $30, $40, and $50 a month, according to the grade, is allowed.

All grades receive longevity pay, 10 per cent. in addition to the regular salary for every five years up to 40 per cent. after twenty years' service.

The tenure of office is permanent. Officers traveling under orders are allowed actual expenses.

For invitation to appear before the board of examiners, address "Surgeon General, Public Health Service, Washington, D. C."

